# Feasibility of a Markerless Motion Capture System for Balance Function Assessment in Children with Cerebral Palsy

**DOI:** 10.3390/s25185911

**Published:** 2025-09-21

**Authors:** Xiaoxia Yan, Nichola Wilson, Chengyan Sun, Yanxin Zhang

**Affiliations:** 1 Department of Exercise Sciences, University of Auckland, Auckland 1010, New Zealand; xyan250@aucklanduni.ac.nz; 2 Department of Surgery, University of Auckland, Auckland 1010, New Zealand; n.wilson@auckland.ac.nz; 3 Department of Surgery, Shanghai Eber Hospital, Shanghai 200000, China; drsuncy@163.com

**Keywords:** cerebral palsy, postural control, center of mass, validity, markerless motion capture

## Abstract

Children with cerebral palsy (CP) have impaired standing balance ability, caused by increased muscle tone, muscle weakness, and joint deformity. It is necessary to investigate standing balance for children with CP. Compared with postural stability assessment using force plates, OpenCap has the potential to be used utilized as a cost-effective standing balance assessment tool, providing details about the center of mass (CoM) and joint angles. This study aims to evaluate the feasibility of using OpenCap for standing balance assessment in children with CP by (i) examining the validity of OpenCap-based CoM parameters against force plate center of pressure (CoP) measures and (ii) exploring associations between joint angles and CoM displacement. Twenty-two children with CP completed standing balance trials on a force plate-based balance tester while two smartphones recorded synchronized videos for OpenCap processing. For the correlation between CoM parameters and CoP parameters, Pearson’s R values were from 0.39 to 0.94 between the two systems. After correcting the CoM parameters, the R^2^ values ranged from 0.98 to 1.00. Regarding the relationship between the joint angles and CoM, maximum and minimum sagittal angles in the ankle were corrected with CoM displacement along the x-axis. These findings suggest that OpenCap may be a potential, cost-effective tool for standing balance assessment in children with CP.

## 1. Introduction

Cerebral palsy (CP) is a permanent neurological disorder that manifests in early childhood [1], characterized by persistent impairments, including abnormal muscle tone, muscle weakness, and deficits in sensory, visual, and cognitive systems [2]. Increased muscle tone and muscle weakness represent positive and negative features of the upper motor neuron syndrome, respectively [3]. These symptoms often result in deficits in selective motor control, restricting joint mobility, symmetry, and balance [1]. Consequently, individuals with CP often experience considerable challenges in motor control and posture maintenance, especially in standing balance [4].

Previous studies using force plates have identified deficits in postural control among children with CP [2,3,5,6] significantly limiting daily activities and reducing quality of life [7]. Effective standing balance is critical in performing daily tasks and functional movements, as it reduces fall risks and provides the foundation for developing advanced motor skills [8]. Enhanced standing balance improves gait, muscle strength, coordination, and physical, psychological, and social participation.

Balance control, a fundamental motor function, impacts daily activities in children with CP. The displacement of the center of mass (CoM) during standing tests indicates the level of postural stability of these children. Research suggests that independently standing children with CP exhibit increased postural sway [7] and atypical CoM trajectories [9], primarily due to spastic joint movement [10,11]. Although CoM measurement provides valuable insights into compensatory strategies for maintaining an upright stance [12], the clinical assessment is complicated due to the precise measurement requirements for joint coordinates and segmental weights. This complexity motivates research into the relationships between CoM and kinematics, potentially informing the development of targeted interventions to improve balance control and reduce fall risks in children with CP [13].

In clinical practice, static balance is primarily evaluated using two primary biomechanical parameters: the center of pressure (CoP) and the CoM. CoP, defined as the point where the vertical ground reaction force vector intersects the support surface, reflects whole-body sway and forces required to maintain the CoM within the base of support [14]. Force plates, which detect pressure changes resulting from shifts in the body weight, measure CoP with high precision and reliability [15], thus serving as the gold standard for balance evaluation. A recent study in healthy older adults found moderate to strong associations between CoP measures from force plates and CoM measures derived from inertial measurement units (IMUs), covering total sway path, velocity, and the area of the 95% confidence ellipse [16].

Markerless motion capture systems, such as OpenCap, provide a promising alternative for assessing CoM directly. These systems use computer vision and machine learning algorithms to accurately track body movements and extract kinematic data, including joint angles and segmental positions, with reasonable accuracy [17,18]. Although markerless motion capture has demonstrated promise in measuring various kinematic parameters [17], its application and validation in clinical populations, especially children with CP, remain limited.

This study investigates the validity of markerless motion capture for calculating CoM parameters by comparing it with force plate CoP parameters during standing in children with CP. Additionally, it aims to explore the relationship between the joint angles and the displacement of CoM. It was hypothesized that CoM parameters calculated by OpenCap systems are correlated with CoP parameters by the balance tester. It was also hypothesized that relationships between joint angles and CoM would be plane independent, with sagittal ankle angles associated with sagittal CoM displacement.

## 2. Materials and Methods

### 2.1. Study Design and Data Sources

The Auckland Health Research Ethics Committee and the local institutional review board approved this prospective observational study. Children were recruited from Shanghai Eber Hospital between September 2024 and December 2024.

### 2.2. Participants

This study included twenty-two children with CP, aged 3 to 12 years, who were classified at Gross Motor Functional Classification System (GMFCS) Level І to Ш and had sufficient functional and cognitive ability to complete the balance test. Exclusion criteria were: uncontrolled epilepsy, visual impairment, and inability to follow instructions. Each participant and their guardian provided written informed consent. Their demographic details are reported in Table 1. Assistive devices and orthoses were removed during testing.

### 2.3. Experimental Setups

Data were collected in the Department of Rehabilitation Treatment at Eber Hospital. The standard clinic rehabilitation assessment room was equipped with a markerless motion capture system (MLS) consisting of two iPhone smartphones, a force plate system (FPS) with a balance tester. Following OpenCap’s recommended two-camera setup, smartphones were mounted on tripods at a distance of 2 m from the participant and 1.1 m in height. The cameras were positioned at an angle of approximately 20–30° relative to the sagittal plane in front of the participants. The balance tester (Balance-B, NCC, Beijing, China) is a computerized device designed to evaluate coordination and balance using four piezoelectric sensors to track the CoM projection [19].

**Figure 1:**
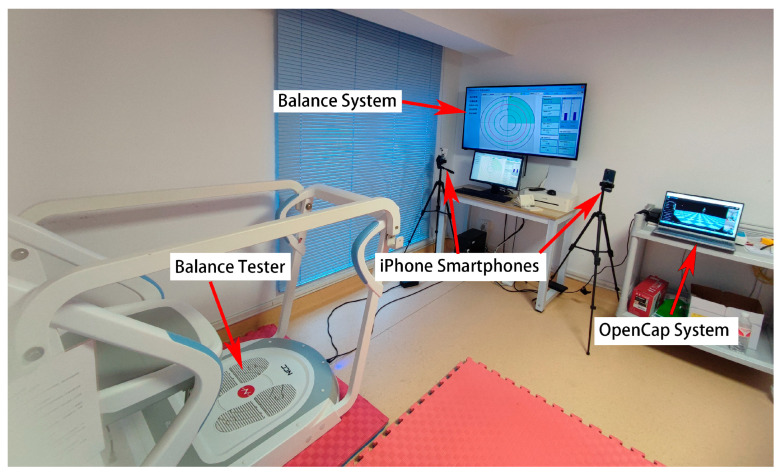
Diagram of rehabilitation assessment room, featuring a balance tester, two displays for the balance system, two iPhone Smartphones, and a laptop for the OpenCap system.

**Figure 2:**
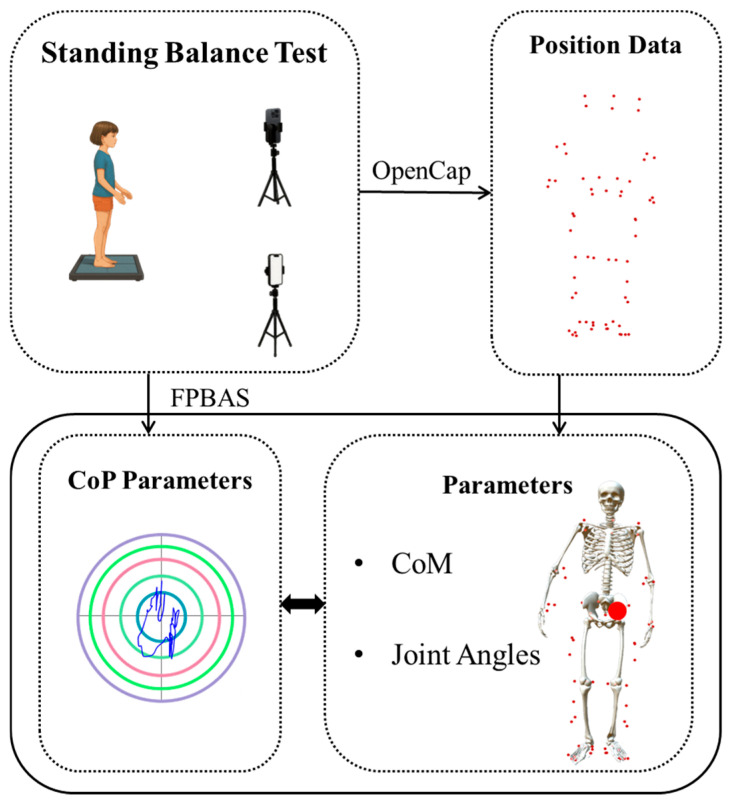
Experimental setup for OpenCap balance assessment and the variables used in analysis. Two smartphones record quietly standing on a force plate-based analysis system (FPBAS) while OpenCap tracks virtual anatomical landmarks, including head, neck, shoulder, elbow, wrist, pelvis, hip, knee, ankle, and toe, to compute joint angles and CoM. The large red dot in the Parameters panel indicates the simulated CoM location. In the CoP Parameters panel, the irregular blue curve represents the CoP trajectory, while the concentric colored circles illustrate reference ranges.

Synchronous data acquisition was managed by OpenCap’s proprietary software (Version 1.0.1), which was developed at Stanford University. The methods for gait data capture with this system, along with its validity, have been previously documented [17]. Prior to data collection, participants with cerebral palsy were provided with a familiarization period with the equipment and experimental procedures. Each assessment session involved a neutral calibration phase followed by a 30 s standing test, performed barefoot. Participants were suggested to maintain visual focus on a wall-mounted screen at eye level under consistent indoor lightning and without holding on handrails, which served only as a safety measure. Any contact with the handrails invalidated the test. Figure 1 illustrates the laboratory setup, and Figure 2 shows the variables used in the analysis.

After synchronized videos were captured using two iOS devices (iPhone 14), OpenCap processed the data to estimate 3D movement dynamics through several steps. Initially, 2D key points were extracted from multi-view videos using pose estimation algorithms (e.g., HRNet). These key points were synchronized across video streams by cross-correlating their velocities. The synchronized 2D key points were then triangulated to derive 3D positions, which were transformed into a comprehensive 3D anatomical marker set using long short-term memory (LSTM) neural networks trained on the motion capture dataset. Finally, inverse kinematics and a musculoskeletal model with biomechanical constraints were applied to calculate 3D kinematics from marker trajectories [17]. Model parameters were adjusted for children with CP by modifying joint constraints in the local deployment. In the OpenSim musculoskeletal model (LaiUhlrich2022) the joint topology was not modified, and no additional degrees of freedom (DOFs) were introduced. Only the constraints at the knee, hip, and ankle joints were relaxed, without the addition of new DOFs.

### 2.4. Data Processing and Analysis

The CoP data were obtained using the vertical force vector measured by the force transducer. Several parameters were extracted from the balance reports, including total shake track (cm), x-axis and y-axis trajectories (cm), x-axis and y-axis shifts (cm), total swaying speed (cm/s), and directional speeds (cm/s) along the x-axis and y-axis [2].

Anatomical landmark trajectories captured by the OpenCap were processed using a fourth-order, zero-lag Butterworth low-pass filter with a 30 Hz cut-off frequency for non-gait trials [17]. In all coordinate systems of each bone segment, the x-axis points anteriorly, the z-axis points to the right, and the y-axis points superiorly when the joint angles are 0° [20].

Segmental CoM coordinates were further calculated using segmental weights and the relative positions of segmental CoM based on segmental length. Whole-body CoM for each participant was calculated as the sum of the segmental CoM values [21]. Balance parameters were computed using the following equations [2]:

(1) $$ShakeTrack=\;\sum_{i=1}^{n-1}\sqrt{{\left(x_{i+1}-x_{i}\right)}^{2}+{\left(z_{i+1}-z_{i}\right)}^{2}}$$

(2) $${ShakeTrack}_{X}=\sum_{i=1}^{n-1}|x_{i+1}-x_{i}|$$

(3) $${ShakeTrack}_{Z}=\sum_{i=1}^{n-1}|z_{i+1}-z_{i}|$$

where $x_{i}$ and $z_{i}$ represented the x-axis and z-axis coordinates in a frame, respectively. Shake track is defined as the total displacement across frames.

Speeds were calculated as follows:

(4) $$\mathrm{Speed}=\frac{ShakeTrack}{time}$$

(5) $${Speed}_{x\;}=\frac{\;{ShakeTrack}_{x}}{time}$$

(6) $${Speed}_{z}=\frac{{ShakeTrack}_{z}}{time}$$

The calculation of the CoM shift was recorded with the last position in the test.

Linear regression analysis was performed to evaluate the correlations between CoM and CoP. Separate regression models were developed for x-axis and y-axis displacements to determine how CoM displacement influenced CoP variation. The coefficient of determination (R^2^), ranging from 0 to 1, quantified the proportion of CoP variance explained by CoM displacement. For each paired parameter, ordinary least-squares models were fitted:

$${Parameter}_{CoP}=\beta_{0}+\beta_{1}\times {Parameter}_{CoM}$$

Reported outputs include slope ($\beta_{1},\;$ 95% CI), intercept ($\beta_{0}$, 95% CI), R^2^, and root mean square error (RMSE).

Participants were divided into three groups based on the involvement pattern of the lower limbs: left hemiplegia, right hemiplegia, and diplegia. Joint angles of each participant were normalized to 100% of the time dimension. The normalized symmetry index (${SI}_{norm}$) was calculated following established methods from the previous studies [22,23].

To investigate the relationship between joint angles and CoM displacement for children with spastic diplegia (*n* = 18), segmental movement angles (ankle, knee, hip, pelvis, trunk, upper limbs) in the sagittal plane were analyzed, including the maximum flexion/extension angles, the range of CoM along the z-axis. Multivariate regression analysis was performed, with CoM parameters as the dependent variables and joint angles as independent variables. Regression coefficients (β_n_) indicated the changes in CoM displacement for each unit change in the corresponding joint angle, holding all other angles constant. All statistical analyses were performed using MATLAB R2023b (MathWorks, Natick, MA, USA).

## 3. Results

### 3.1. Correlation Analysis Between CoM and CoP

Pearson correlation analysis was conducted to investigate the relationships between CoP and CoM across various balance parameters (Table 2). Total shake track demonstrated a strong positive correlation (R = 0.81, *p* < 0.01). Similarly, shake tracks along the x-axis and z-axis showed strong correlations (R = 0.82, *p* < 0.01; R = 0.74, *p* < 0.01, respectively). The z-axis CoM shift exhibited the strongest correlation (R = 0.94, *p* < 0.01), indicating excellent agreement with CoP measurements, while the x-axis CoM shift showed a moderate correlation (R = 0.39). Total speed and x-axis speed demonstrated moderate positive correlations (R = 0.66, *p* < 0.01 for both), and Z-axis speed showed a moderate positive correlation (R = 0.51, *p* = 0.02).

Ordinary least-squares regressions mapping ${Parameter}_{CoM}$ to ${Parameter}_{CoP}$ (Table 2) showed moderate fits for the shake track metrics (R^2^ = 0.55–0.68; RMSE = 3.20–4.80) and a strong fit for Shift-z (β_1_ = 66.80 [55.20, 78.40]; β_0_ = 1.59 [1.24, 1.94]; R^2^ = 0.88; RMSE = 0.79). Shift-x displayed a weak association (β_1_ = 42.83 [−3.72, 89.38]; R^2^ = 0.16; RMSE = 2.44). Speed metrics showed lower explanatory power (R^2^ = 0.26–0.44; RMSE = 0.16–0.22).

### 3.2. Kinematic Symmetry Index Analysis

Kinematic and symmetry analyses (Table 3) revealed notable movement asymmetry in children with diplegia. Right ankle plantarflexion (8.16 ± 0.89) demonstrated greater asymmetry than the left (6.33 ± 0.87), with a significant normalized symmetry index ${SI}_{norm}$ of −24.44% (SD = 9.83%). Similar asymmetry patterns were observed in knee flexion and hip flexion. In contrast, hip adduction was relatively symmetric, with similar angles on the right (−3.27 ± 0.69) and left (−6.79 ± 0.83). The pelvis was tilted anteriorly, with evaluation on the right side. Upper limb movements, including shoulder flexion, shoulder adduction, and elbow flexion, were symmetric, though the right side exhibited slightly greater displacement.

Children with left hemiplegia exhibited symmetric and asymmetric patterns. Ankle plantarflexion was greater on the left, while knee and hip flexion showed slight asymmetries: knee flexion was more pronounced on the left, and hip flexion on the right. Hip adduction showed significant asymmetry, with the left hip more abducted. The pelvis was tilted anteriorly, with the right side higher. Arm and elbow flexion were nearly symmetric; however, left-side shoulder abduction was greater.

Children with right hemiplegia exhibited kinematic asymmetries. Ankle plantarflexion presented minimal asymmetry, while knee and hip flexion showed greater values on the right (19.10 ± 2.44 and 18.10 ± 3.15, respectively) than on the left (12.35 ± 2.73 and 13.81 ± 3.39), corresponding to ${SI}_{norm}$ of −3.16% and −12.64%. Hip adduction was more pronounced on the right (−6.27 ± 1.52) than on the left (13.65 ± 27.12). The pelvis was tilted anteriorly, with evaluation on the left. Upper limb analysis revealed reduced right shoulder flexion (−2.79 ± 3.63 vs. 5.95 ± 2.41), as well as greater right-side arm abduction and elbow flexion (−20.08 ± 3.28 and 75.22 ± 17.90) compared to the left (−10.33 ± 3.11 and 38.19 ± 10.19), resulting in the ${SI}_{norm}$ of 7.02% and 22.11%, respectively.

### 3.3. Multiple Linear Regression of CoM Displacement and Joint Angle

In children with diplegia, multiple linear regression analysis identified associations between joint angles and the CoM displacement along the x, y, and z axes. As presented in Table 4, the x-axis CoM displacement was positively associated with maximum sagittal knee and minimum sagittal ankle angles and the minimum coronal arm angles, indicating these variables contribute to increased lateral movement of the CoM. Similarly, for the z-axis, sagittal ankle angles, sagittal knee angles, and coronal kinematics of the shoulder and elbow showed positive correlations with displacement, indicating a rightward CoM shift.

## 4. Discussion

This study evaluated the feasibility of using OpenCap to calculate CoM and joint angles in standing balance tests in children with CP. Additionally, it examined the relationship between joint angles and CoM displacement. The results demonstrated that OpenCap-based balance parameters exhibited moderate to strong correlations with force plate measurements, supporting the validity for assessing standing balance.

Previous studies have shown acceptable accuracy of OpenCap in measuring joint angles in gait analysis [17,18]. This study is the first to extract CoM parameters from OpenCap in standing balance, providing preliminary evidence of its potential for screening and monitoring balance development. Although correlations with force plate measurements were moderate, OpenCap shows promise as a practical and accessible alternative for balance assessment in clinical settings. The system provides accurate keypoint tracking [18], enabling the analysis of kinematic symmetry and CoM deviations, which are associated with movement efficiency and balance deficits [24,25]. Its user-friendly and markerless design also facilitates remote assessment, making it a cost-effective solution for community or home healthcare settings.

This study found that moderate to strong correlations between force plate-based CoP parameters and OpenCap-derived CoM parameters for the children with CP, aligning with Ferrari et al. (2024) [16], who reported comparable associations using IMU-based CoM in healthy older adults, despite differences in population and sensing modality. The convergence across cohorts and technologies supports the expected biomechanical linkage between CoM state and CoP, and provides criterion evidence that markerless video–based CoM estimates can capture clinically relevant balance variability in the children with CP. However, correlation does not imply interchangeability because of device and potential fixed biases may persist. In summary, the results in this study indicate that OpenCap-derived CoM metrics offer feasible and informative parameters for balance assessments.

Consistent with previous research, this study observed increased hip and knee flexion in children with CP [26], which may be attributable to characteristic muscle activation patterns in those with crouch posture. The accuracy of joint angle estimation from markerless systems in pathological populations remains a concern. A previous study [18] noted that measurement errors can reach up to 11 degrees. Compared to prior studies [27,28], the diplegia cohort in this study had similar hip flexion, reduced knee flexion, and ankle plantarflexion, possibly related to the different surgical histories and measurement models. Pronounced kinematic asymmetries were also identified, potentially contributing to compromised motor function, increased risk of injury [29], and decreased energy efficiency. Compensatory upper limb movements, such as increased shoulder flexion, abduction, and elbow flexion, were also evident in standing balance tasks.

Multiple linear regression analysis revealed significant associations between joint angles and CoM displacement in children with diplegia, underscoring the importance of joint movement in balance control. Limited ankle mobility increased CoM displacement in both AP and ML directions. Kinematic asymmetries and the influence of upper limb movement on CoM displacement were also observed. These findings provide valuable insights for developing targeted rehabilitation strategies to improve balance by reducing CoM displacement.

This study has several limitations. First, the sample size limits subgroup analyses and the cross-sectional design. Second, CoM was estimated using OpenCap without concurrent marker-based validation in this study. Accordingly, a marker-based comparison should be performed to interpret CoM parameters in future work. Third, the balance tester system did not export raw CoP time series, restricting analyses to summary sway metrics and preventing within-trial CoM-CoP coupling analyses as described by Winter [30]. Fourth, all trials were performed with participants focusing on a static visual target; our findings represent that visually assisted stance and sway may be larger under reduced or variable visual cues, because children with CP often rely on vision, and an external focus can facilitate more automatic postural control [31,32]. Fifth, we did not measure lower-limb strength or passive range of motion, factors associated with standing balance in CP [33]. Incorporating these biomechanical parameters in future research could further clarify contributors to balance impairment in this population. Finally, this study used a standard musculoskeletal model, which was developed based on the anthropometrics of typically developing individuals. Children with CP might have altered segment lengths and muscle volumes, which may reduce the accuracy of estimations.

## 5. Conclusions

This study primarily evaluated the validity of OpenCap for CoM-based balance parameters in children with CP. CoM parameters showed moderate to strong correspondence with force plate CoP outcomes, supporting the feasibility of using markerless motion capture to quantify postural sway. Additionally, multiple linear regression analysis revealed that sagittal ankle and coronal arm movements significantly influence CoM displacement in children with CP. These findings suggest that OpenCap has the potential to be a cost-effective and portable tool for balance evaluation. Future research should focus on developing algorithms to improve skeletal tracking accuracy.

## Figures and Tables

**Table 1:** Demographic data for the children with CP, values are means (SD).

Type of CP	Age (y)	Weight (kg)	Height (m)	Gender M/F	Surgery	GMFCS І/II/Ш
Hemiplegia (L)	3	17.75	1	2/0	OS/SDR + OS: 1/1	0/0/2
Hemiplegia (R)	9	25.5	1.3	0/2	SDR + OS: 2	0/1/1
Diplegia	6.39	21.89	1.12	12/6	SDR + OS/OS/SDR:8/5/2 OS + CPS/no surgery:2/1	2/9/7
Total	6.32 (3.92)	21.85 (9.31)	1.12 (0.18)	14/8	SDR + OS/OS:11/6 SDR/OS + CPS: 2/2 No surgery: 1	2/10/10

CPS: cervical perivascular sympathectomy. OS: orthopedic surgery. SDR: selective dorsal rhizotomy.

**Table 2:** Results in the correlation analysis and linear regression analysis between CoM and CoP parameters.

Indicator	Pearson Correlation Analysis		Linear Regression Analysis			
	R	*p*	$\beta_{1}$ (95%CI)	$\beta_{0}$ (95%CI)	R^2^	RMSE
Shake Track	0.81	<0.01	8.26, (5.51, 11.01)	−5.03 (−12.49, 2.42)	0.66	4.80
Shake Track x	0.82	<0.01	6.33, (4.30, 8.37)	−2.92 (−7.98, 2.14)	0.68	3.20
Shake Track z	0.74	<0.01	15.53, (8.98, 22.09)	−3.74 (−9.04, 1.56)	0.55	3.73
Shift-x	0.39	0.07	42.83, (−3.72, 89.38)	2.87 (−0.80, 6.54)	0.16	2.44
Shift-z	0.94	<0.01	66.80, (55.20, 78.40)	1.59 (1.24, 1.94)	0.88	0.79
Speed Ave	0.66	<0.01	7.30, (3.43, 11.16)	−0.10 (−0.45, 0.26)	0.44	0.22
Speed x	0.66	<0.01	6.05, (2.88, 9.23)	−0.08 (−0.35, 0.12)	0.44	0.16
Speed z	0.51	0.02	9.36 (2.02, 16.7)	0.02 (−0.19, 0.23)	0.26	0.16

Ave: average, $\beta_{1}$: slope, $\beta_{0}$: intercept, RMSE: root mean square error, R^2^: coefficient of determination. CI: confidence interval.

**Table 3:** Summary of joint angles and symmetry.

Type	Kinematics	Right (°)	Left (°)	${SI}_{norm}$ (%)
Diplegia	Ankle-plantarflexion	8.16 (0.89)	6.33 (0.87)	−2.44 (9.83)
	Knee-flexion	17.52 (0.81)	19.40 (1.04)	−20.79 (14.02)
	Hip-flexion	11.73 (0.95)	13.92 (1.08)	−4.42 (9.36)
	Hip-adduction	−3.27 (0.69)	−6.79 (0.83)	−0.39 (32.33)
	Pelvis-tilt	−5.13 (0.81)		/
	Pelvis-list	−0.57 (0.47)		/
	Arm-flexion	5.90 (2.22)	5.73 (2.30)	4.50 (9.92)
	Arm-adduction	−9.04 (1.41)	−8.45 (0.94)	13.13 (15.95)
	Elbow-flexion	42.58 (4.82)	42.30 (4.41)	1.41 (15.10)
Hemiplegia (Left)	Ankle-plantarflexion	3.64 (4.10)	4.11 (3.00)	12.37 (16.16)
	Knee-flexion	22.01 (5.83)	25.72 (3.32)	−2.54 (19.29)
	Hip-flexion	23.33 (4.21)	22.12 (4.92)	2.33 (12.37)
	Hip-adduction	−3.90 (3.30)	−11.33 (3.56)	9.48 (28.53)
	Pelvis-tilt	−9.85 (2.29)		/
	Pelvis-list	−0.62 (1.90)		/
	Arm-flexion	12.81 (11.36)	18.12 (17.60)	0.98 (10.17)
	Arm-adduction	−5.90 (4.07)	−8.44 (5.88)	15.38 (14.02)
	Elbow-flexion	30.55 (12.01)	27.35 (8.40)	0.52 (12.26)
Hemiplegia (Right)	Ankle-plantarflexion	2.40 (3.35)	2.07 (1.62)	−0.79 (14.39)
	Knee-flexion	19.10 (2.44)	12.35 (2.73)	−3.16 (15.69)
	Hip-flexion	18.10 (3.15)	13.81 (3.39)	−12.64 (9.05)
	Hip-adduction	−6.27 (1.52)	0.35 (1.42)	13.65 (27.12)
	Pelvis-tilt	−8.58 (1.87)		/
	Pelvis-list	1.78 (1.05)		/
	Arm-flexion	−2.79 (3.63)	5.95 (2.41)	−18.37 (13.31)
	Arm-adduction	−20.08 (3.28)	−10.33 (3.11)	7.02 (29.16)
	Elbow-flexion	75.22 (17.90)	38.79 (10.19)	22.11 (26.43)

${SI}_{norm}$: normalized symmetry index. Values are mean (SD).

**Table 4:** Summary of regression coefficients in diplegic patients. (10^−3^).

	CoM Range X	CoM Range Y	CoM Range Z
Hip sag-min, R/L	0.0/−0.5	0.0/−0.1	0.0/−1.7
Hip sag-max, R/L	0.9/0.0	0.3/0.0	0.6/0.0
Knee sag-min, R/L	1.0/0.0	−0.1/0.0	−1.4/0.0
Knee sag-max, R/L	0.6/1.3	0.3/0.1	0.9/1.6
Ankle sag-min, R/L	0.3/0.2	0.9/−1.1	1.1/0.2
Ankle sag-max, R/L	0.2/0.0	−2.7/0.0	−3.2/0.0
Shoulder sag-min, R/L	0.0/0.5	0.0/−0.4	0.0/−0.26
Shoulder sag-max, R/L	1.3/0.8	−0.3/2.0	−1.2/1.3
Shoulder cor-min, R/L	−1.0/3.6	2.1/−1.6	−0.7/0.2
Shoulder cor-max, R/L	0.0/1.8	0.0/−2.6	0.0/−2.4
Elbow sag-min, R/L	1.1/0.0	1.5/−0.2	−0.4/−0.3
Elbow sag-max, R/L	−1.2/1.2	−0.3/−0.1	0.9/−0.9

cor: coronal angles, adduction, and abduction. L: left side. max: maximum. min: minimum. R: right side. sag: sagittal angles, flexion and extension in hip, knee, and elbow; plantarflexion and dorsiflexion in ankle. CoM: center of mass.

## Data Availability

Due to ethical and privacy constraints, data are available upon reasonable request from the corresponding author.

## Abbreviations

The following abbreviations are used in this manuscript:

CP: cerebral palsy; CoP: center of pressure; CoM: center of mass; ICC: intraclass correlation coefficient; UCM: uncontrolled manifold; GMFCS: Gross Motor Functional Classification System; CPS: cervical perivascular sympathectomy; OS: orthopedic surgery; SDR: selective dorsal rhizotomy; MLS: markerless motion capture system; FPS: force plate system.
